# Lulu Regulates Shroom-Induced Apical Constriction during Neural Tube Closure

**DOI:** 10.1371/journal.pone.0081854

**Published:** 2013-11-25

**Authors:** Chih-Wen Chu, Emma Gerstenzang, Olga Ossipova, Sergei Y. Sokol

**Affiliations:** Department of Developmental and Regenerative Biology, Icahn School of Medicine at Mount Sinai, New York, New York, United States of America; UC Irvine, United States of America

## Abstract

Apical constriction is an essential cell behavior during neural tube closure, but its underlying mechanisms are not fully understood. Lulu, or EPB4.1l5, is a FERM domain protein that has been implicated in apical constriction and actomyosin contractility in mouse embryos and cultured cells. Interference with the function of Lulu in *Xenopus* embryos by a specific antisense morpholino oligonucleotide or a carboxy-terminal fragment of Lulu impaired apical constriction during neural plate hinge formation. This effect was likely due to lack of actomyosin contractility in superficial neuroectodermal cells. By contrast, overexpression of Lulu RNA in embryonic ectoderm cells triggered ectopic apico-basal elongation and apical constriction, accompanied by the apical recruitment of F-actin. Depletion of endogenous Lulu disrupted the localization and activity of Shroom3, a PDZ-containing actin-binding protein that has also been implicated in apical constriction. Furthermore, Lulu and Shroom3 RNAs cooperated in triggering ectopic apical constriction in embryonic ectoderm. Our findings reveal that Lulu is essential for Shroom3-dependent apical constriction during vertebrate neural tube closure.

## Introduction

Apical constriction is a behavior of polarized epithelial cells that is essential for epithelial tissue folding during many morphogenetic processes, such as neural tube formation, gut invagination or dorsal closure [1]. This phenomenon is characterized by cell elongation along the apico-basal axis and contraction of the apical surface, leading to the wedge-like shape [2]. At the onset of vertebrate neural tube closure, cells at certain locations within the neural plate undergo apical constriction and form hinge points allowing the neural plate to bend toward the midline [3–5]. Since neural tube defects (NTDs) are among the most common human birth abnormalities with more than 200 genes implicated in this process [6,7], analysis of the molecular mechanisms underlying apical constriction is a focus of embryological studies aimed at prevention of birth defects.

In the current model of apical constriction, microtubule and actomyosin contractile activity play critical roles in cell elongation and apical surface shrinkage respectively [2]. The assembly of apico-basally oriented microtubule arrays is required for cells to elongate along the apico-basal axis, which is an important step underlying apical constriction during neural tube closure [8–10]. On the other hand, Rho-associated kinase (ROCK) - dependent activation of non-muscle myosin II results in apical enrichment of the contractile actomyosin cytoskeleton that generates mechanical forces leading to the constriction of the apical surface [8,11–13]. One of the proteins implicated in this process is Lulu (also known as Epb41l5), a FERM (Four-point-one, Ezrin, Radixin, Moesin)-domain-containing protein that is required for neural plate morphogenesis in mice [14,15]. Lulu has been reported to drive apical constriction in MDCK cells in a ROCK-dependent manner, and the neural tube of Lulu mutant mice exhibits mislocalized actomyosin network [14,16]; however, whether Lulu functions in neural plate morphogenesis by facilitating apical constriction is unclear. Moreover, how Lulu activates the ROCK pathway to trigger apical constriction is largely unknown. 

To study the in vivo function of Lulu during neural tube closure, we chose to use *Xenopus* embryos, which are easily manipulated at relevant developmental stages and in which apical constriction can be induced ectopically, in the absence of other patterning processes [3]. We show that depletion of Lulu by antisense morpholino oligonucleotides (MO) leads to defective neural fold elevation and hingepoint formation, due to failed apical constriction and apical accumulation of actomyosin contractile complexes in the neuroepithelium. RNAs encoding the *Xenopus* homolog of Lulu induced ectopic apical constriction in ectodermal cells, and this activity was inhibited by a carboxy-terminal fragment of Lulu. Furthermore, we find that Lulu promotes the apical accumulation of Shroom3, an actin-binding protein that triggers apical constriction by locally activating ROCK [13,17,18], and cooperates with Shroom3 to induce apical constriction. Together, these findings indicate that Lulu contributes to neural fold formation by regulating Shroom3 activity and apical constriction. 

## Results

### Lulu is required for neural fold formation in Xenopus embryos

**Figure 1:**
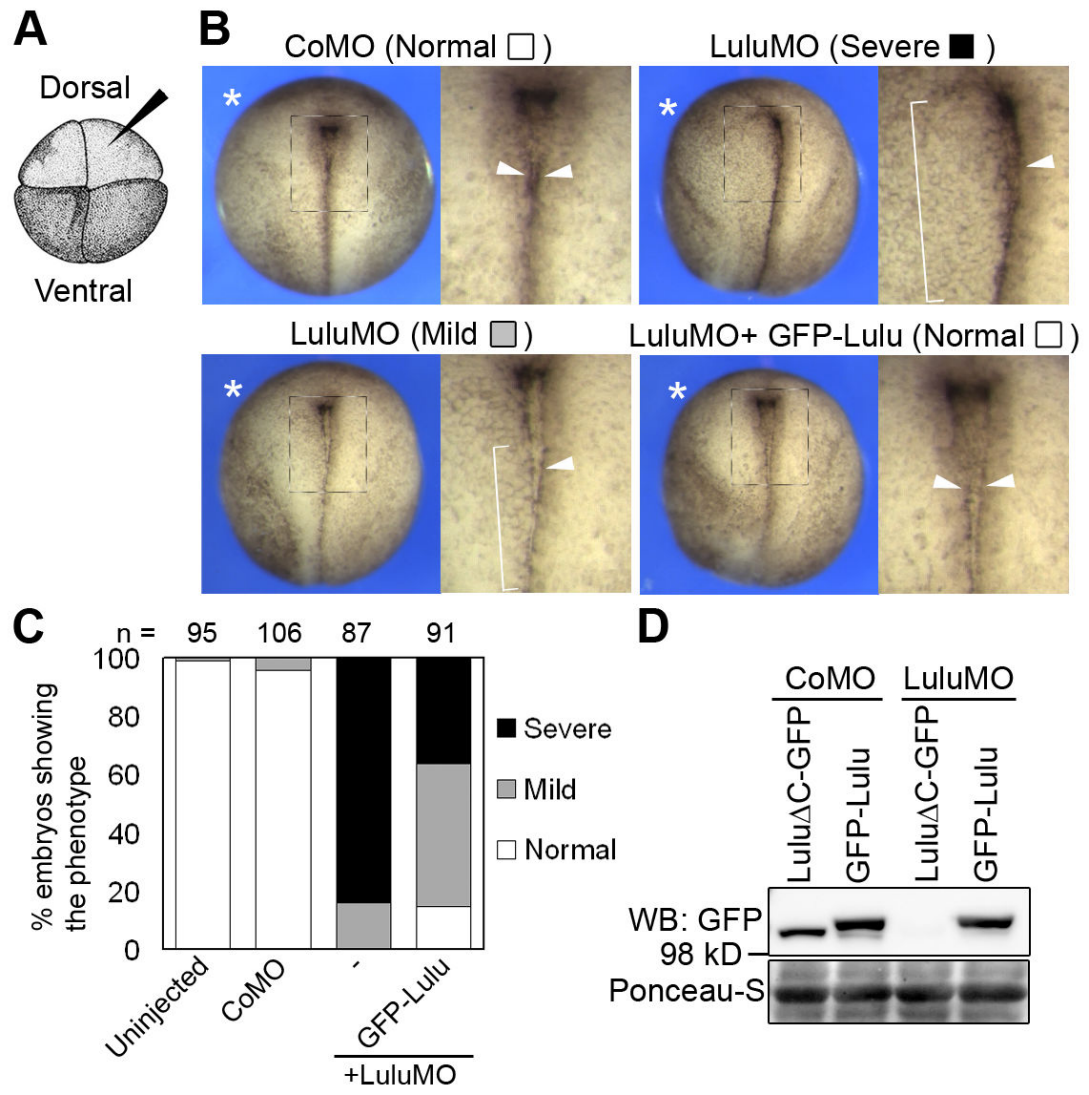
Lulu is required for neural plate hinge point formation. (A) A four-cell stage embryo viewing from the animal pole. Injections were done in one dorsal animal blastomere (arrowhead). (B) Top views of stage 18 embryos unilaterally injected with indicated morpholinos (MOs, 20 ng) and GFP-Lulu mRNA (25 pg). Asterisks mark the injected side. Squared areas are magnified on the right of each panel. Arrowheads point to the hinge, visible as a pigment line, and brackets indicate weakening or disappearance of the hinge. For quantification of defects, lack of the pigment line was scored as “severe”, and weak or discontinuous pigment line was scored as “mild.” (C) Frequencies of defects shown in (B). (D) Lysates from embryos injected with indicated mRNAs and MOs were subjected to western blot. Ponceau-S staining shows loading.

In mammals, two alternatively spliced isoforms of Lulu have been described that share the FERM and the FA (FERM adjacent) domains and differ in their C-terminal regions [14]. In *Xenopus laevis*, our search of NCBI databases identified a Lulu homologue with 75% identity to the short isoform of human Lulu (data not shown). To explore the functions of Lulu during embryogenesis, we depleted endogenous Lulu by injecting an anti-sense MO, which specifically targets the 5’ non-coding region of Lulu (LuluMO, see Materials and Methods). After injections into a single dorsal animal blastomere of four-cell embryo, LuluMO did not cause any visible defects during gastrulation. At later stages, however, LuluMO-injected, but not control MO (CoMO)-injected embryos developed NTDs (Figures 1A-C). Detailed examination of these embryos revealed broadened and flattened neural fold at the LuluMO-injected side (Figure 1B, asterisk), as compared to the uninjected side and embryos injected with CoMO. In addition, LuluMO suppressed the formation of hingepoints (Figure 1B, brackets), which normally appear as densely pigmented lines along the neural fold [3,19] (Figure 1B, arrowheads). LuluMO was effectively blocking the translation of LuluΔC-GFP RNA, which contains MO target sequence, but not of GFP-Lulu RNA, a construct lacking MO target sequence (Figure 1D). When co-injected with LuluMO, GFP-Lulu RNA partially rescued the NTDs caused by LuluMO (Figure 1B and 1C), attesting to MO specificity. When injected on its own, GFP-Lulu RNA did not have detectable effects on neural plate morphology at this dose (data not shown). These results indicate that Lulu is essential for *Xenopus* neural tube closure.

### Depletion of Lulu disrupts apical accumulation of actomyosin in neuroepithelial cells

**Figure 2:**
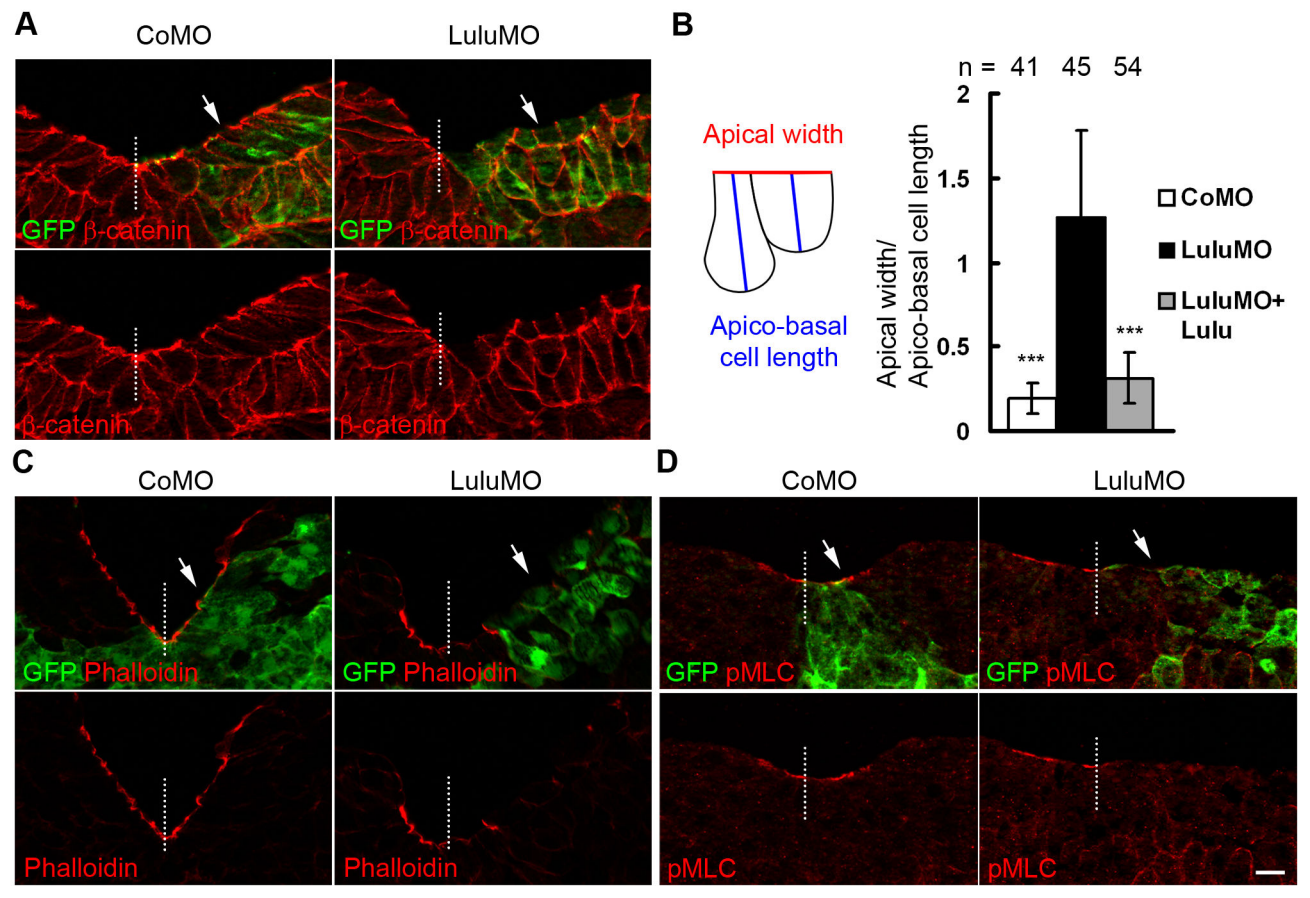
Depletion of Lulu disrupts apical constriction in neuroectoderm. (A, C-D) Transverse sections of the neural plate of stage 17 embryos unilaterally injected with 250 pg of GFP-CAAX mRNA and indicated MOs were stained to visualize GFP and (A) β-catenin, (C) F-actin by phalloidin, (D) phosphorylated myosin light chain (pMLC). Representative images are shown. In (C) and (D), exposure was adjusted to avoid oversaturation of apical staining. Arrows mark the injected side. Dashed lines mark midline. Scale bar: 20 µm. (B) Ratios of apical width to apico-basal cell length of superficial neuroepithelial cells were measured and compared. Up to five cells adjacent to midline were measured per section. Means +/- s. d. are shown. ***: p<0.0001, as compared to the LuluMO group.

Neural plate hingepoints indicate the location of cells undergoing apical constriction, a major driving force for neural plate bending [3]. Failure of hingepoint formation in LuluMO-injected embryos implies that apical constriction is disrupted. To further investigate the effect of Lulu depletion at the cellular level, embryos co-injected with MOs and GFP-CAAX mRNAs were cultured until stage 17 and cryo-sectioned. Whereas apico-basal elongation and apical constriction of superficial cells were apparent at the uninjected and control MO-injected side and resulted in the elevated neural fold, LuluMO suppressed apical constriction and neural fold elevation (Figure 2A, arrows). To quantify the effect of MOs on cell shape change, we compared ratios of apical width to apico-basal cell length for the superficial cells at the neural fold stage, and observed much higher ratio (i. e., inhibited apical constriction) in LuluMO-injected embryos as compared to CoMO-injected controls (Figure 2B). Co-injection of GFP-Lulu with LuluMO significantly reduced the ratio of apical width to apico-basal cell length (Figure 2B), indicating that the apical constriction defect is specific to Lulu depletion. These observations reveal that Lulu is essential for apical constriction of neuroepithelial cells.

F-actin, myosin IIB and phosphorylated myosin light chain (pMLC) are enriched apically in the neuroepithelium and are known to contribute to apical constriction and neural tube closure [11,14,20]. Since the disruption of *Lulu* in mice leads to mislocalized F-actin and myosin IIB [14], we examined the effect of LuluMO on F-actin and pMLC distribution in transverse sections of the neural plate at midneurula stages. Both F-actin and pMLC were highly enriched at the apical surface of control superficial neuroepithelial cells (Figure 2C and 2D, arrowheads), but both markers were significantly decreased in LuluMO-injected cells (Figure 2C and 2D, arrows). By contrast, depletion of Lulu did not affect the basolateral staining of β-catenin and ZO1 (Figure 2A and data not shown), further confirming specificity. These results suggest that *Xenopus* Lulu is involved in apical accumulation and assembly of actomyosin contractile complexes during neural tube closure. 

### Lulu induces ectopic apical constriction and apical accumulation of F-actin in ectodermal cells

**Figure 3:**
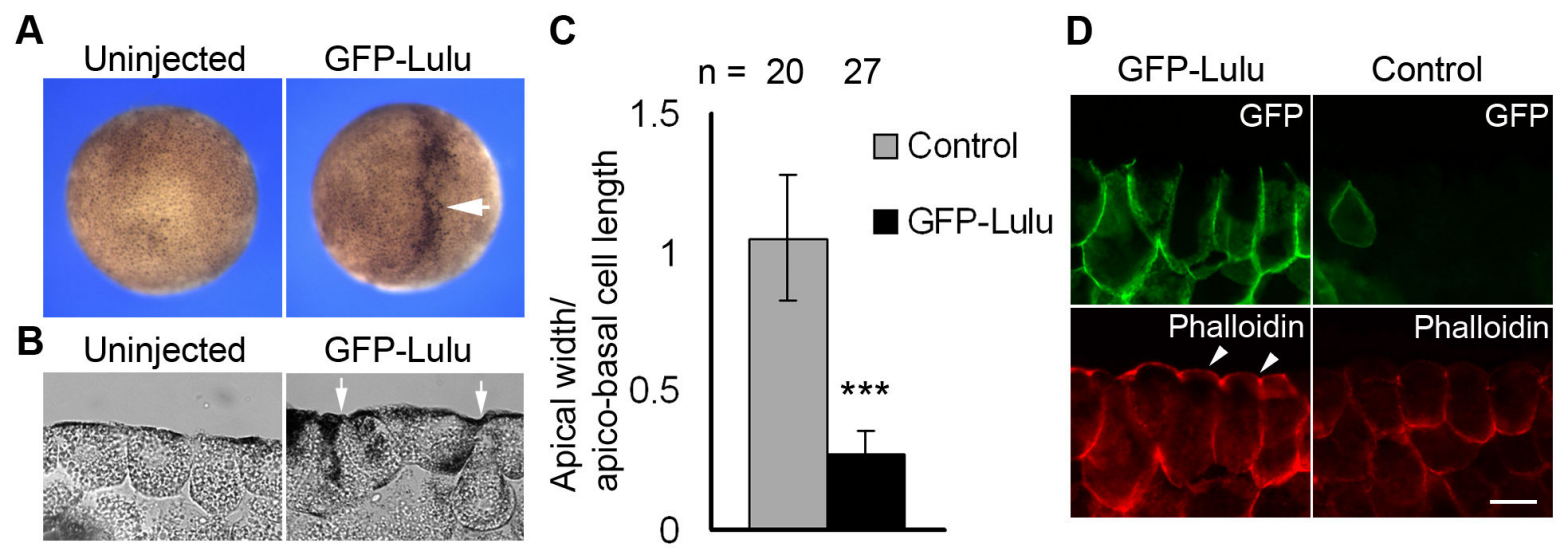
Overexpression of Lulu induces ectopic apical constriction in vivo. (A) Top views of stage 9 embryos uninjected or injected with 250 pg of GFP-Lulu mRNA. The arrow indicates hyper-pigmented cells. (B) Sections of stage 9 embryos uninjected or injected with GFP-Lulu mRNA. Arrows point to constricting cells. Scale bar: 20 μm. (C) The ratio of apical width to apico-basal cell length of superficial ectodermal cells is shown. Means +/- s. d. are shown. ***: p<0.0001. (D) Sections of stage 9 embryos injected with GFP-Lulu mRNA were stained with anti-GFP antibody and phalloidin. Superficial ectodermal cells are shown. Note the increase of apical and junctional staining of phalloidin in cells expressing GFP-Lulu (arrowheads). Scale bar: 20 μm.

To gain more insight into the functions of Lulu in vivo, mRNAs encoding GFP-Lulu were injected into animal pole of two blastomeres of four-cell embryos. At stage 9, GFP-Lulu induced ectopic apical constriction and increased pigmentation that often accompanies apical constriction [3] (Figure 3A, arrow). Cross-sections of the injected embryos confirmed the wedged shape of constricting cells, in contrast to the cuboidal shape of control cells (Figure 3B, arrows; Figure 3C). Moreover, expression of GFP-Lulu, which localized predominantly to the basolateral membrane domain, induced ectopic F-actin at the apical surface and junctions of superficial cells (Figure 3D, arrowheads). Taken together, these observations document the ability of Lulu to induce ectopic apical constriction *in vivo*.

### The FERM and FA domains are necessary for Lulu subcellular localization and are essential for apical constriction

**Figure 4:**
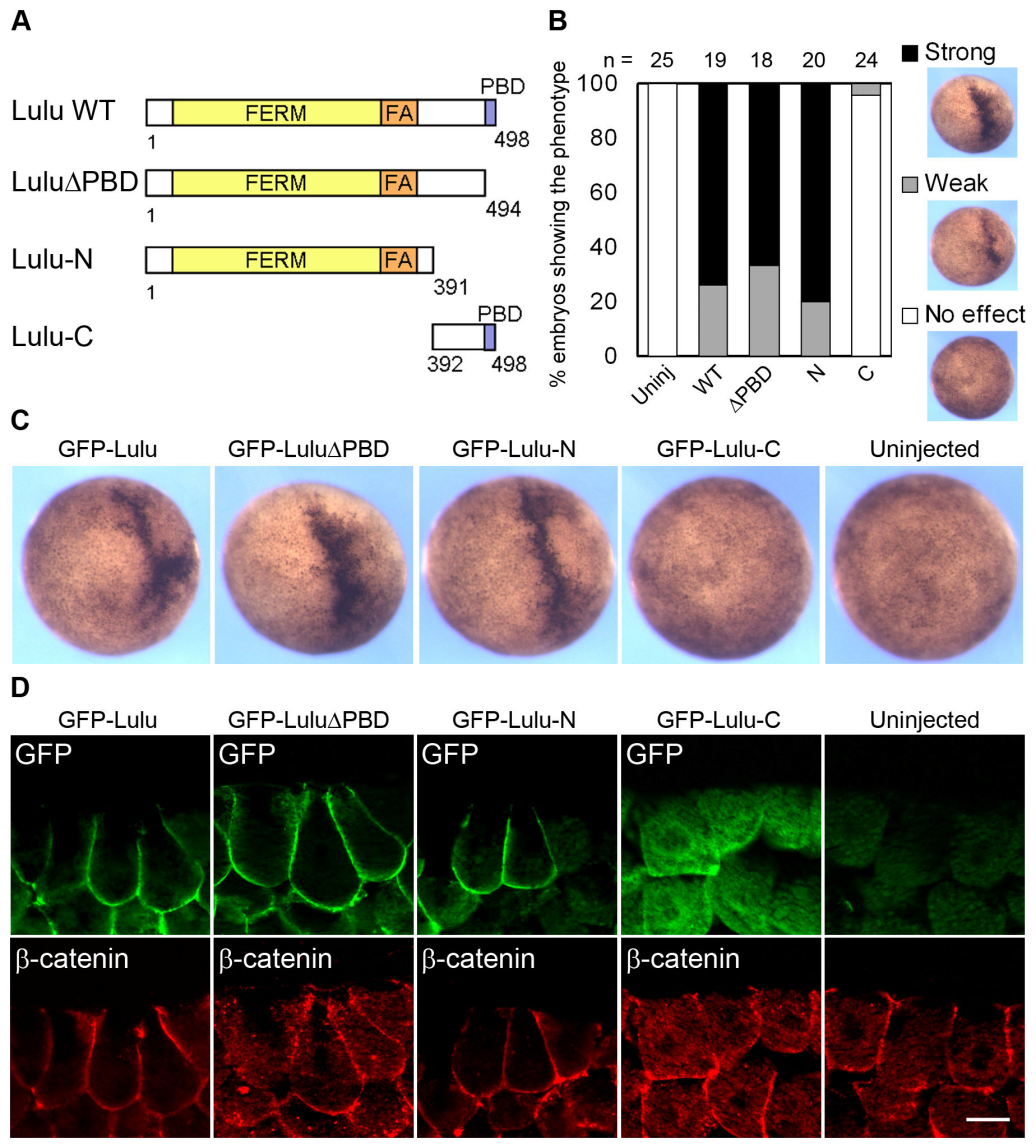
The FERM and FA domains of Lulu are necessary for the induction of apical constriction. (A) Schematic illustration of various deletion mutants of Lulu. Amino acid numbers are indicated. (B) Embryos were injected with 250 pg of mRNAs encoding indicated Lulu mutants. Percentage of the embryos showing ectopic hyper-pigmentation at stage 9 was shown. Phenotypes were scored based on the degree of pigmentation increase, and representative images are shown on the right side. (C) Top views of stage 9 embryos injected with indicated mRNAs. (D) Sections of stage 9 embryos injected with indicated mRNAs were stained with anti-GFP and anti-β-catenin antibodies. Superficial ectodermal cells are shown. Note the change of cell shape shown by the staining of β-catenin. Scale bar: 20 μm.

To map the domains of Lulu responsible for apical constriction, we made several deletion mutants and tested their capability of inducing apical constriction in ectodermal cells (Figure 4A). The mutant lacking the PDZ-binding domain (LuluΔPBD) or most of the C-terminal region (Lulu-N) was able to induce hyper-pigmentation and apical constriction as potently as the full-length protein (Figure 4B-D). This finding is consistent with the reported ability of the FERM and FA domains of mouse Lulu to induce apical constriction in MDCK cells [16]. On the other hand, Lulu-C, which has no N-terminal FERM and FA domain, was unable to induce apical constriction, and it also failed to localize to the basolateral cortex (Figure 4B-D). These findings indicate that the FERM and FA domain of Lulu are necessary and sufficient for Lulu basolateral targeting and for its ability to induce apical constriction.

### The carboxy-terminal region of Lulu functions in a dominant-negative manner

**Figure 5:**
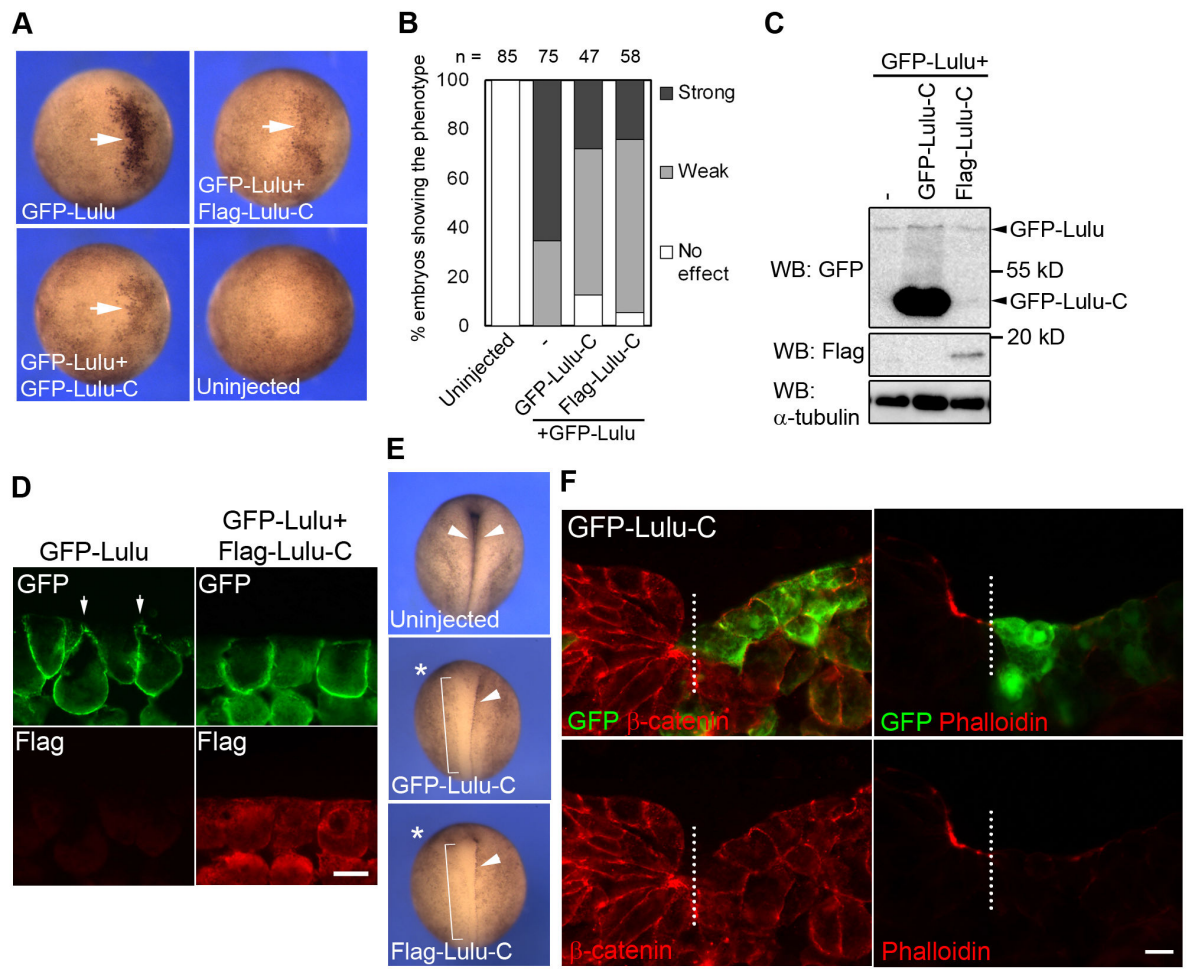
The carboxy-terminal fragment of Lulu functions as a dominant-negative mutant. (A) Top views of stage 9 embryos injected with indicated mRNAs. 100 pg of GFP-Lulu mRNA and 5 ng of GFP-Lulu-C or Flag-Lulu-C mRNAs were injected. Arrows point to ectopic hyper-pigmentation. (B) Percentage of the embryos showing ectopic hyper-pigmentation. Phenotypes were scored as described in Figure 4 legend. (C) Lysates from embryos injected with indicated mRNAs were subjected to Western Blot. α-tubulin serves as the loading control. (D) Sections of stage 9 embryos injected with indicated mRNAs were stained with anti-GFP and anti-flag antibodies. Superficial ectodermal cells are shown. Note the change of cell shape shown by the staining of GFP. Arrows indicate constricting cells. (E) Top views of stage 17 embryos uninjected or unilaterally injected with 5 ng of Lulu-C mRNA. Asterisks mark the injected side. Arrowheads point to the hinge of neural plate, and brackets indicate disappearance of the hinge. (F) Transverse sections of the neural plate of stage 17 embryos, unilaterally injected with GFP-Lulu-C mRNAs, were stained to visualize GFP, β-catenin, and F-actin. Dashed lines mark midline. Scale bar: 20 μm.

Lulu has been shown to bind p120-catenin via its N-terminus and paxillin via its C-terminus, indicating that Lulu may act as an adaptor protein enhancing the association between these proteins [15]. Given that Lulu-C contains a PDZ-binding domain (PBD), we hypothesize that overexpression of Lulu-C may sequester those PDZ-containing Lulu-binding proteins, thereby interfering with the function of endogenous Lulu. To test this hypothesis, GFP-Lulu RNA was co-injected with GFP or Flag-tagged Lulu-C RNA, and the extent of apical constriction phenotype was examined at stage 9. Lulu-induced pigmentation and apical constriction were reduced by co-expression of Lulu-C (Figures 5A-B). Lulu protein levels were comparable in embryo lysates in the presence or absence of Lulu-C (Figure 5C), suggesting that the inhibition of apical constriction is not due to altered protein amount. Moreover, immunofluorescent staining revealed that Flag-Lulu-C inhibited GFP-Lulu-induced apical constriction without changing the localization of GFP-Lulu (Figure 5D). Taken together, these findings indicate that Lulu-C inhibits the effects of exogenous Lulu, and may thus function in a dominant-negative manner.

We next examined the effect of Lulu-C on neural tube closure. Injection of GFP-Lulu-C RNA into one dorsal-animal blastomere of four-cell stage embryos inhibited hingepoint formation and neural tube closure in 60 % of the injected embryos (Figure 5E, brackets), similar to the effect of LuluMO (Figure 1B). Immunostaining of transverse sections of stage 17 embryos confirmed that Lulu-C-expressing neuroepithelial cells did not undergo apical constriction, and apical accumulation of F-actin was abolished as compared to the uninjected side (Figure 5F). The similarity of the phenotypes induced by Lulu-C overexpression and LuluMO further supports an essential role of Lulu in neural tube closure. 

### Lulu is required for Shroom3-induced apical constriction in ectoderm cells

**Figure 6:**
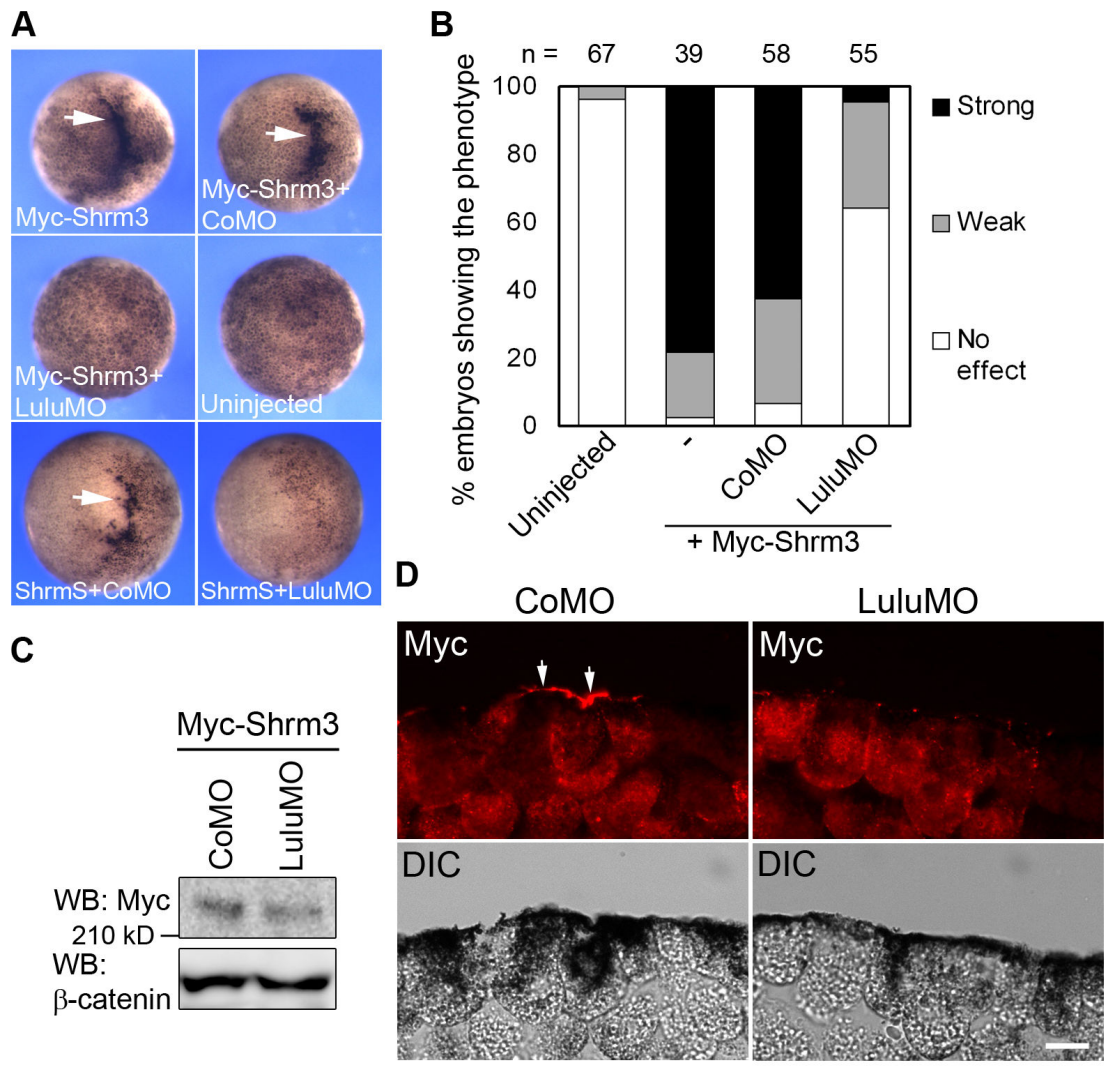
Lulu activity is required for Shroom3 to induce apical constriction. (A) Top views of stage 9 embryos injected with indicated mRNAs and MOs. 50 pg of Myc-Shroom3 (Myc-Shrm3) or ShroomS (ShrmS) mRNAs and 20 ng of MOs were injected. Arrows point to ectopic hyper-pigmentation. (B) Percentage of the embryos showing the constriction phenotype. Phenotypes were scored as described in Figure 4 legend. (C) Lysates from embryos injected with indicated mRNAs and MOs were subjected to western blotting. α-tubulin serves as the loading control. (D) Sections of stage 9 embryos injected with indicated mRNAs and MOs were stained with anti-Myc antibodies. Superficial ectodermal cells are shown. Arrows point to constricting cells. Scale bar: 20 μm.

Shroom3, an activator of the ROCK-myosin II signaling pathway, has been reported to induce apical constriction during neural tube closure in mice and Xenopus [3,13,17,18]. To assess a possible connection between Shroom3 and Lulu, we wanted to test whether Shroom3 activity depends on Lulu. Myc-Shroom3 RNA was co-injected with either CoMO or LuluMO into animal pole of early embryos and monitored ectopic apical constriction at stage 9. Myc-Shroom3 co-injected with CoMO induced profound apical constriction in 62% of the injected embryos, while LuluMO significantly inhibited this phenotype to less than 5% (Figure 6A and 6B). ShroomS, the short isoform of Shroom3 that has a different amino-terminal sequence, also failed to induce apical constriction when co-injected with LuluMO (Figure 6A). This further supports functional specificity of LuluMO. Similar levels of Myc-Shroom3 were expressed in embryo lysates injected with CoMO or LuluMO (Figure 6C), suggesting that this inhibition is unlikely due to effects on protein translation or stability. 

### Lulu regulates Shroom3 accumulation at the apical surface during apical constriction

Based on the finding that Shroom3 activity depends on Lulu, we hypothesized that Lulu acts together with Shroom3 to induce apical constriction, possibly by promoting the apical localization and activity of Shroom3. We therefore investigated whether Shroom3 protein localization is affected in the absence of Lulu on cross-sections of late blastula embryos co-injected with Myc-Shroom3 and CoMO or LuluMO. Myc-Shroom3 was enriched at the apical surface in 31.8% of the injected cells when co-injected with CoMO (n = 45; Figure 6D, arrows). In contrast, when endogenous Lulu was depleted by LuluMO, Myc-Shroom3 was localized to apical junctions and cytoplasm, with 1.9% of the injected cells showing strong Myc staining at the apical surface (n = 55; Figure 6D). These results imply that Lulu is required for the proper localization of Shroom3 during apical constriction.

**Figure 7:**
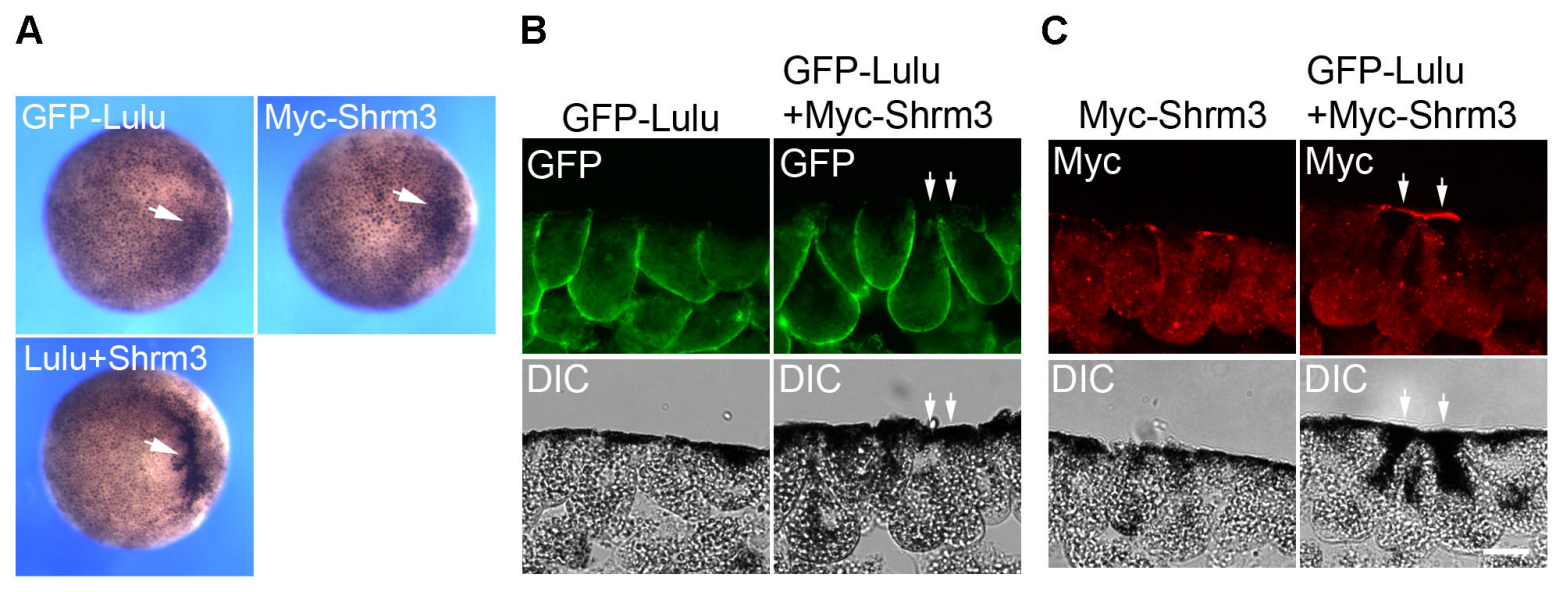
Lulu synergizes with Shroom3 to induce apical constriction. (A) Top views of stage 9 embryos injected with indicated mRNAs. 25 pg of GFP-Lulu mRNA and 10 pg of Myc-Shroom3 (Shrm3) mRNA were injected. Arrows point to ectopic hyper-pigmentation. (B, C) Sections of stage 9 embryos injected with indicated mRNAs were stained with anti-Myc (B) and anti-GFP antibodies (C). Superficial ectodermal cells are shown. Arrows point to constricting cells. Scale bar: 20 μm.

Next, suboptimal amounts of GFP-Lulu and Myc-Shroom3 RNAs were co-injected into animal pole of the embryos. When injected alone, GFP-Lulu and Myc-Shroom3 induced weak pigmentation increase in only 20% and 16% of the embryos at stage 9, respectively; however, when they were co-injected, strong pigmentation and apical constriction were seen in 83% of the embryos (Figure 7A-C, arrows), suggesting that Lulu synergizes with Shroom3 to induce apical constriction. Immunostaining of the embryo sections revealed that GFP-Lulu remained basolaterally localized in the presence of Myc-Shroom3 (Figure 7B). On the other hand, while Myc-Shroom3 was localized to apical junctions and cytoplasm at this dose (6.1% of the injected cells showing intense apical staining of Myc, n = 74), co-expression of GFP-Lulu led to apical accumulation of Myc-Shroom3 in 36.4% of the injected cells (n = 60; Figure 7C, arrows). These findings indicate that Lulu regulates Shroom3 localization and cooperates with Shroom3 to induce apical constriction. 

## Discussion

This study shows that interfering with the function of Lulu by MO-mediated depletion or overexpression of Lulu-C RNA impairs neural tube closure in *Xenopus* embryos. These observations are consistent with the phenotypes of Lulu mutant mice and suggest a conserved function of Lulu in vertebrate neurulation and apical constriction [14,15]. Interestingly, while depletion of Lulu prevented apical accumulation of F-actin and pMLC in *Xenopus* embryos, the neural plate of Lulu mutant mice shows mislocalized F-actin and myosin II without significant decrease in protein levels [14]. Similarly, while disruption of Lulu homologues in zebrafish *moe* and *Drosophila yurt* mutants causes reduction or mislocalization of Crumbs, an apical polarity determinant, mouse Lulu mutant embryos reveal no change in Crumbs localization [14,21–23]. These observations suggest that other proteins act together with Lulu in regulating actomyosin complexes and apico-basal cell polarity in mouse embryos. One candidate gene that is known to function redundantly with Lulu in mammalian cells is *Ehm2* (also known as *Epb41l4b* or *Lulu2*). Ehm2 is required for the establishment of the actomyosin contractile ring along apical junctions, and it can also induce apical constriction in MDCK cells [16,24]. Further studies of Ehm2 and other FERM domain proteins that are related to Lulu would contribute to the understanding of the roles of FERM proteins in morphogenesis.

Protein-protein interactions mediated by the association between PBD and PDZ domain are commonly seen in signaling pathways that regulate actin reorganization [25]. Although not necessary for inducing ectopic apical constriction, the carboxy-terminal PBD has an important functional role, as it is required for Lulu to rescue the defects of the zebrafish *moe* mutant (Figure 4) [16,26]. Our findings reveal that overexpression of Lulu-C caused neural fold formation defects similar to the phenotypes of Lulu depletion (Figure 1B and 5E), suggesting that Lulu-C acts in a dominant-negative manner, possibly by sequestering endogenous Lulu-interacting proteins. Identification and characterization of these proteins would help gain further insights into how Lulu functions.

What are the mechanisms underlying Lulu-induced apical constriction? In Lulu knock-out mice, the level of E-cadherin is elevated, suggesting that Lulu promotes E-cadherin turnover during gastrulation [15]. Although E-cadherin is present largely in non-neural ectoderm, it has been implicated in neural tube closure, possibly by regulating actomyosin networks [27]. Depletion of Lulu in epidermis did not affect E-cadherin staining pattern (data not shown), arguing against the possibility that Lulu regulates neural tube closure indirectly by affecting E-cadherin in non-neural ectoderm.

Although ROCK is required for Lulu-induced apical constriction in MDCK cells, the molecular connection between ROCK and Lulu is still obscure [16]. We observed that the apical constriction triggered by GFP-Lulu is attenuated by a dominant-negative RhoA mutant (data not shown), supporting the hypothesis that active RhoA is involved in Lulu-induced apical constriction. Moreover, we uncovered that Shroom3 localization and activity are regulated by Lulu, making Shroom3 a good candidate to link Lulu to ROCK activation. By contrast, a dominant-negative mutant of Shroom3 [3] failed to block Lulu-induced apical constriction in embryonic ectodermal cells (data not shown), consistent with the lack of effect of Shroom3 siRNA on Lulu-dependent apical constriction in MDCK cells [16]. Furthermore, Shroom3 activity and subcellular localization are regulated by active RhoA and Trio, suggesting that Lulu may promote apical accumulation of Shroom3 by activating Trio or other RhoA GEFs [18,28]. In fact, p114RhoGEF can drive apical constriction downstream of Ehm2, whereas PDZ-RhoGEF is required for apical constriction during chick neural tube closure [24,29,30]. Thus, while Lulu is likely to activate specific RhoA GEFs during neural tube closure, their identities remain to be revealed in future studies. 

## Materials and Methods

### Ethics Statement

This study was carried out in strict accordance with the recommendations in the Guide for the Care and Use of Laboratory Animals of the National Institutes of Health. The protocol 04-1295 was approved by the IACUC of the Icahn School of Medicine at Mount Sinai**.**

### Plasmids and morpholino oligonucleotides

Xenopus Lulu cDNA clone (accession number BC055968) was purchased from Open Biosystems (Huntsville, AL). The coding sequence was amplified by PCR and subcloned into pXT7-GFP and pCS2-flag to generate GFP-Lulu and flag-Lulu, respectively. An EcoRI-SacI fragment was excised from the original cDNA clone and ligated with GFP and pCS2 to generate LuluΔC-GFP, which contains the 5′UTR sequence targeted by LuluMO. The pCS2-GFP-CAAX construct was previously described [31]. The mouse Shroom3 constructs (ShroomL, referred here as Shroom3, and ShroomS) are gifts from Phil Soriano [17]. The Shroom3 coding sequence was subcloned into pCS2 with an N-terminal Myc tag. Capped mRNAs were in vitro synthesized using mMessage mMachine kit (Ambion, Austin, TX). For MO injection, control MO (CoMO) (5’-GCTTCAGCTAGTGAC ACATGCAT-3’) and Lulu-specific MO (LuluMO) (5’-ACATCTTTAAGCTGG GTGGCAGCGG-3’) were purchased from Gene Tools (Philomath, OR). Cloning details are available upon request.

### Embryo Culture and Manipulation

In vitro fertilization, microinjection and embryo culture were carried out as described previously [32]. Developmental stage of the embryos was determined as described [33]. Microinjections were done by injecting 10 nl of solutions into the animal pole of four-cell stage embryos. For the inhibition of Shroom3 activity by LuluMO, microinjections were done in the animal pole of two-cell stage embryos. Images of whole embryo were taken using Leica stereomicroscope and Openlab software.

### Western Blot Analysis

Xenopus embryos at stage 10 were lysed in the buffer containing 50 mM Tris-HCl pH 7.6, 50 mM NaCl, 1 mM EDTA, 1% Triton X-100, 10 mM NaF, 1 mM Na_3_VO_4_, 25 mM β-glycerol phosphate and 1 mM PMSF. After centrifugation at 15000g for 5 minutes, the supernatant was subjected to SDS-PAGE. Western blot was carried out as described previously [34]. Staining signals of anti-α-tubulin antibody (clone B512, Sigma) or Ponceau S solution (Sigma) were used as the loading control. Images were captured using LAS-3000 luminescent analyzer system (Fujifilm).

### Fluorescence Microscopy and Quantification

Embryos were manually devitellinized and fixed with Dent's fixative (80% methanol: 20% DMSO) for 2 hours at room temperature and rehydrated with PBS. Fixation for staining of phalloidin or phospho-myosin light chain was described previously [8]. Cryosectioning and indirect immunofluorescence staining were performed on cross-sections as described previously [32]. The following primary antibodies were used: mouse anti-GFP (B-2, Santa Cruz), rabbit anti-GFP (A6455, Invitrogen), rabbit anti-β-catenin (c2206, Sigma), rabbit anti-phospho-S20-myosin light chain (ab2480, Abcam), mouse anti-Myc (9E10, Roche), mouse anti-flag (M2, Sigma). For F-actin staining, Alexa Fluor 568-conjugated phalloidin (4 units/ml, Invitrogen) was used. Secondary antibodies were Alexa Fluor 488-conjugated (Invitrogen) or Cy3-conjugated (Jackson ImmunoResearch). Images were captured using a Zeiss AxioImager microscope with the Apotome attachment. Image processing and measurements of cell morphology were done on images of sections stained with β-catenin antibodies using the AxioVision software (Zeiss). All measurements were collected from at least three embryos. Apical width was defined as the distance between apical cell junctions. Apico-basal cell length was defined as the distance between the midpoints of the apical and the basal domains. For Figure 2B, up to five superficial neuroectodermal cells adjacent to the midline were measured. For Figure 3C, sections including both uninjected cells and GFP-Lulu-expressing cells were used, and five to seven cells in each group were measured per image. Statistical analysis was performed using Microsoft Excel software, and p values were determined using Student's t-test. 
